# Confirmation of negative urine culture status after appropriate antibiotic treatment prior to endourological stone procedures: Is it really necessary?

**DOI:** 10.1007/s00240-023-01524-5

**Published:** 2024-02-08

**Authors:** Cahit Sahin, Resul Sobay, Alper Asik, Emre Burak Sahinler, Salih Yildirim, Kamil Kul, Kemal Sarica

**Affiliations:** 1 Department of Urology, Health Sciences University, Sancaktepe Sehit Prof. Dr. Ilhan Varank Research and Training Hospital, Istanbul, Turkey; 2 Department of Urology, Health Sciences University, Umraniye Research and Training Hospital, Istanbul, Turkey; 3 Department of Urology, Biruni University, Faculty of Medicine, Istanbul, Turkey

**Keywords:** Urine culture test, Flexible ureteroscopy, Negative urine culture, Urinary tract infection, Post-operative infective complication

## Abstract

To evaluate the necessity of confirmation for a negative urine culture test outcome after an appropriate antibiotic regimen for urinary tract infection (UTI) prior to endoscopic stone removal procedures. 170 cases receiving an appropriate antibiotic treatment for culture proven UTI based on test outcomes before endoscopic stone removal were evaluated in two groups: Group 1 (*n* = 85) Patients in whom a second urine culture test was performed to ensure “negative urine culture” status prior to the procedures after receiving antibiotic therapy and Group 2 (*n* = 85). Patients receiving the same antibiotic therapy without any additional urine culture test before the procedures. Cases were comparatively evaluated with respect to the statistical significance of post-operative infective complications (fever, sepsis), duration of hospital stay and readmission rates during early post-operative period. Our findings demonstrated no significant difference regarding the rate of infective complications (presence of fever, incidence of septic findings), hospitalization period and readmission rates between the two groups. Although the presence of a negative urine status has been confirmed by urine culture test in group 1 cases, no additional urine culture test was performed with this aim in group 2 cases (negative urine culture was confirmed only with urinalysis) and the outcomes regarding the infective problems were found to be similiar. Our current findings indicate that a second urine culture test may not be a “must” if the patients receive an appropriate antibiotic regimen based on the sensitivity test outcomes for a reasonable time period.

## Introduction

As an endemic disease in some parts of the world, urolithiasis is an important worldwide healthcare problem based on its recurrent nature [1].Regarding the management principles,with the help of marked advances in instrument technology and new treatment concepts, most stones are managed with minimal invasive treatment modalities among which extracorporeal shockwave lithotripsy (SWL); flexible ureteroscopy (fURS) and percutaneous nephrolithotomy (PCNL) are the commonly applied techniques [2, 3].

Of these alternatives, fURS is the preferred modality for both ureteric and renal stones sizing < 2 cm. As a safe and less invasive method than PCNL, fURS is associated with minor complications varying from 9 to 25% [4, 5] and limited major complications (< 1%) [6]. Among these complications the infective problems are the most crucial ones where post-operative fever occurs in 7.1%, UTI in 8.1% and sepsis in 5.4% of all cases managed with this modality [7–10]. On the other hand, PCNL is the preferred treatment modality for large (> 2 cm) stones at one session. However, despite its successful outcomes with overall stone free rates of 75–98%, PCNL is regarded as an invasive approach which could be associated with certain sever complications (bleeding, perforation and sepsis) [2, 6].Regarding the complication profile of these two procedures, infective complications should be regarded as the most crucial ones to keep in mind[2].Evaluation of the published data for two modalities on this aspect demontrates that while fURS could be associated with such infective problems in 0.95–8.1% of the cases [9–12] these values have been reported to be 0.9–16.7 after PCNL [7–15].

Thus, a well planned, rational decision making along with a thorough urine examination seem to be the most approach for safe and successful outcomes. To limit or even prevent such infective complications which may sometimes cause death, all cases should be well evaluated to outline the presence and severity of UTI. In case of a documented infection based on mid-stream urine culture and sensitivity test, UTI should inevitably be treated well to render the patient infection free before such procedures.The presence of leucocytes and/or nitrite in urinalysis will be regarded as reliable indicators for UTI. European Association of Urology (EAU) guidelines recommend to perform a urinary microscopy and/or obtaining a urine culture test before stone removal for an effective antibiotic management [5]. However, both EAU and American Urological Association (AUA) guidelines acknowledge that their recommendations for antibiotic prophylaxis are based on limited evidence regarding the choice of antimicrobial agents, dosages applied, timing and duration of procedure [5, 6, 10].

On the other hand,with respect to the treatment of UTI before such procedures, although no distinct data has been reported to date to emphasize the necessity of a perioperative antibiotic prophylaxis for fURS, the EAU guidelines recommend prophylactic antibiotic administration prior to all such procedures except simple diagnostic URS and distal ureteral stone treatment [16, 17]. For the PCNL procedure, in their original trial Gravas et al. were able to show that a perioperative antibiotic prophylaxis could reduce the rate of post-procedural complications which can even ocur in patients with a negative baseline urine culture [18]. In case of a pre-operative negative urine culture status, a single-dose antibiotic administration was considered to be sufficient [19].However, EAU guidelines state that, few studies could be derived from published literature defining the risk of infection following fURS and PCNL with no clear-cut evidence.

Thus, a careful evaluation of all cases with respect to the presence of UTI and an appropriate antibiotic treatment before endoscopic procedures based on culture sensitivity test outcomes seems to be highly critical. With this approach the likelihood of post-operative infection could be limited or even eliminated to a certain extent. A systematic review demonstrated that performance of a urine culture/sensitivity test is superior to urine analysis in ruling out bacteriuria and should therefore be the reference standard [20]. However, although this approach is being recommended by EAU guidelines, it is not fully outlined whether there is any need for an additional urine culture test to confirm a negative urine culture status after completion of antibiotic treatment before proceeding with stone removal procedure.

In other words, there is an ongoing debate if a second urine culture test is mandatory following a sensitivity based antibiotic treatment for a maximum period of 10 days before any stone removal procedure. Elimination of second urine culture test may simplify the pre-operative diagnostic procedures, shorten the pre-operative phase, lower the laboratory work load and costs.To the best of our knowledge, this is the first study which will outline well the necessity of a second culture test to confirm the urine sterility after antibiotic management. This will let the endourologist to remove the stones without any delay and also let the patient to undergo the procedure without having further stress during this period with a well preserved quality of life.

We aimed to evaluate the role and necessity of confirmation for a “negative urine culture test outcome” after an appropriate antibiotic regimen prior to endoscopic stone removal procedures in cases presenting with culture positive urinary tract infection (UTI).

## Patients and methods

The multicentric study protocol was approved by Hospital ethical commitee. Regarding the selection of the cases for our current study program, files of 5650 patients undergoing fURS and PCNL for kidney Stones between January 2018 and May 2023 (available in hospital database system)were retrospectively evaluated. Following the elimination of the cases not meeting the inclusion criteria, remaining 170 patients with positive urine culture before the these procedures were included. Patients with bilateral and multiple stones, previous stone related procedures, pregnancy,congenital anomalies and solitary kidneys were all excluded. All cases had calcium containing stones. In addition to the patient demographic data such as gender, age, and body mass index (BMI), detailed history, urogenital examination findings, biochemical test outcomes were all recorded. All patients had a urinalysis and urine culture sensitivity test prior to the above mentioned interventions.

fURS was performed by using a single use endoscope (9.0 Fr, HugeMed, China) with the help of ureteral access sheath and Ho-YAG laser under general anesthesia. Standard PCNL procedures were performed with 26 Fr nephroscope (Karl Storz, Tuttlingen, Germany) following percutaneous tract dilatation (Amplatz sheath, Boston Scientific, Natick, MA, USA) until 28–30 Fr. Stones were disintegrated using either Ho-YAG laser unit or an ultrasonic lithotripsy probe (Swiss Lithoclast^®^, EMS Electro Medical System, Nyon, Switzerland). All procedures were performed by the same experienced surgeons in two centers.

Patients (*n*:170) were divided into to subgroups as follows: Group 1 (*n* = 85) Patients with a positive pre-operative urine culture test who were operated after an appropriate antibiotic management based on culture sensitivity test outcomes. Following the completion of the antibiotherapy, the presence of sterile urine status was confirmed with a second urine culture test performed after 48–72 h following the completion of antibiotic management. Thus, patients with a positive pre-operative urine culture was confirmed to have negative urine culture 48–72 h after following the completion of appropriate antibiotic treatment.Group 2 (*n* = 85) Patients with a positive pre-operative urine culture test who were operated after appropriate culture sensitivity outcomes based antibiotherapy and in whom the presence of negative urine culture has not been confirmed with a second urine culture test. Only a urinalysis was performed with a Dipstick test (H800^®^ and FUS200^®^ analytical device (DIRUI, China) in these cases. All cases had a negative urinalysis test outcomes with negative leukocyte esterase activity and/or nitrite [21].

All patients with a positive pre-operative urine culture (significant bacterial growth ≥ 10E5 CFU/ml) received an antibiotic treatment for a maximum of 10 days based on the guidance of antibiotic sensitivity test outcomes with a detailed consultation to the infectious disease department. On the other hand, negative urine culture status has been defined as assessment of ‘‘no growth within 24 or 48 h in the mid-stream clean catch samples that have been collected properly’’. In addition, all cases received an intraoperative single-shot antibiotic prophylaxis with a third generation cephalosporine.The uropathogens identified and tested at the beginning of the tretament were all also susceptible to this antibiotic. Patients were comparatively evaluated with respect to the presence of infectious complications after these procedures.

### Definition of post-operative infective complications

Infectious complications were considered to be present when patients exhibited a fever of > 38 ℃ persisting 48 h [22] and sepsis. Sepsis was defined as the presence of systemic inflammatory response syndrome (SIRS) caused by a suspected infection with 2 or more of the following diagnostic criteria: fever ≥ 38 ℃ or hypothermia ≤ 36.0 ℃, tachycardia > 90 beats/minute, tachypnea > 20 breaths/minute, change of laboratory values (elevation of C reactive protein CRP value > 2.9 mg/dl, leucocytosis > 10.000µL leucocytopenia < 4.000/μL and/or thrombocytopenia < 150.000/μl) [23]. Duration of hospital stay and readmission within the first 30 days were also evaluated between two groups.

### Statistical analyses

Statistical analysis was done by using independent-samples *t* tests to compare continuous variables, such as stone size and operating time and exact Chi-square *t* test to compare the categorical variablesinfectious complications. IBM SPSS Statistics 25 (IBM, Armonk, NY) was used for statistical analysis. The p value was considered significant when *p* value < 0.05.

## Results

**Fig. 1:**
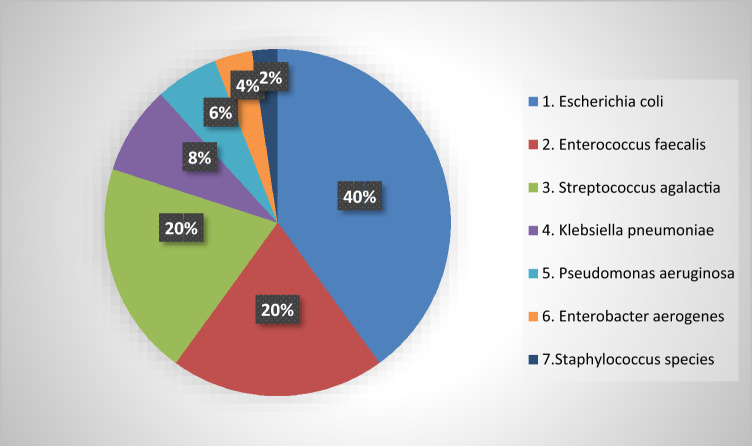
Distribution of isolated microorganisms from the performed urine culture tests

Overall a total of 170 patients were included and while fURS was performed in 147 cases; 23 patients underwent PCNL for kidney stones. The microorganism distribution identified in urine culture tests is shown in Fig. 1.

### Evaluation of our data obtained in both group of cases revealed following findings;

While the mean patient age in 170 patients (101 men and 69 women) was 43.37 ± 14.28, with a mean BMI value of 26.08 ± 3.40 (Table 1); mean size of the stones was 9.38 ± 3.32 in these cases.The overall stone free rate was 143 (84.1%). Average number of hospitalization days was 3.08 ± 3.35, the number of hospital readmissions was 26 (15.3%).There was no statistically significant difference regarding the patient and stone related factors in both group cases Table 1.

**Table 1:** Patient demographicsand stone characteristic in both groups

	Overall (*n* = 170)	Group 1 (*n* = 85)	Group 2 (*n* = 85)	*p*
Gender; *n* (%)				
Male	74 (43.5%)62	38 (44.7%)	36 (42.4%)	0.61
Female	96 (56.5%) 108	56 (65.9%)	52 (61.2%)	
Age; (years)				
Mean ± SD	45.9 ± 13.96	48.04 ± 13.86	43.76 ± 13.81	0.77
BMI; (kg/m2)				
Mean ± SD	26.58 ± 4.17	26.99 ± 4.70	26.17 ± 3.54	0.28
Comorbidity status				
Hypertension; *n* (%)	57 (33.5%)	34 (40.0%)	23 (27.1%)	0.07
Diabetes; *n* (%)	34 (20.0%)	21 (24.7%)	13 (15.3%)	0.12
Stone size, (mm)				
Mean ± SD	16.13 ± 10.98	17.14 ± 12.20	15.20 ± 9.76	0.56
Surgical modality				
PCNL; *n* (%)	23 (13.5%)	12 (14.1%)	11 (12.9%)	
fURS; *n* (%)	147 (86.5%)	67 (45.6%)	80 (54.4%)	
SFR in third month; *n* (%)	143 (84.1%)	73 (85.9%)	70 (82.4%)	0.13

Values were accepted as statistically significant if *p* < 0.05

*PCNL* percutaneous nephrolithotomy, *fURS* Flexible ureteroscopy, *BMI* body mass index, *SFR* stone free rate

With respect to the laboratory evaluation findings, there was no statistically significant difference between two groups on this aspect. While the mean CRP value was 40.48 ± 57.83 in Groıup 1, this value was 38.74 ± 76.04 in Group 2 (*p* = 0.540). In addition, mean serum white blood cell count (WBC) evaluation revealed similar outcomes [9.31 ± 3.87 and 9.94 ± 3.57, respectively (*p* = 0.553)] (Table 2).

**Table 2:** Comparison of postoperative biochemical parameters of infection in both groups

	Overall (*n* = 170)	Group 1 (*n* = 85)	Group 2 (*n* = 85)	*p*
Post-operative				
CRP				
Mean ± SD	39.61 ± 67.36	40.48 ± 57.83	38.74 ± 76.04	0.54
WBC				
Mean ± SD	9.62 ± 3.73	9.31 ± 3.87	9.94 ± 3.57	0.55
Seum creatinine level				
Mean ± SD	1.09 ± 0.68	1.02 ± 0.51	1.16 ± 0.81	0.29
Serum urea level				
Mean ± SD	36.48 ± 22.43	36.83 ± 19.50	36.13 ± 25.14	0.42

Values were accepted as statistically significant if *p* < 0.05

*CRP* C-Reactive protein, *WBC* White blood cell

In terms of post-operative clinical infective complications; sepsis was diagnosed in four cases (4.7%) of Group 1 (2 cases in PCNL group and two cases in fURS group) and how cases (2.4%) of Group 2 (2 cases in fURS groups) without any significant difference between the two groups (*p* = 0.41). Similarly post-operative fever rate was similar in both groups without any significance [20 cases in total; 10 cases in Group 1(11.8%) and 10 cases (11.8%) in Group 2, (*p* = 1.00)] (Table 3).

**Table 3:** Comparison of postoperative clinical parameters of infection in both groups

	Overall (*n* = 170)	Group 1 (*n* = 85)	Group 2 (*n* = 85)	*p*
Postoperative				
Sepsis; *n* (%)	6 (3.5%)	4 (4.7%)	2 (2.4%)	0.41
Fever; *n* (%)	20 (11.8%)	10 (11.8%)	10 (11.8%)	1.00
Hospital stay;days				
Mean ± SD	3.08 ± 3.35	2.58 ± 3.17	3.29 ± 3.45	0.09
Emergency department readmission; *n* (%)	26 (15.3%)	14 (16.5%)	12 (14.1%)	0.67

Values were accepted as statistically significant if *p* < 0.05

Lastly, both the mean duration of hospitalization (*p* = 0.095) and emergency department readmission rates (16.5 vs 14.1%, respectively) were similar in both groups without any statistically significant difference (*p* = 0.670) (Table 3).

## Discussion

Current minimally invasive management options for upper urinary tract stones include URS and PCNL [2, 3]. Although ureteroscopic stone removal procedures are being preferred mainly for medium sized stones (1–2 cm), PCNL is the treatment of choice for the successful treatment of large (> 2 cm) stones. Related with this issue, technological developments and new concepts have expanded the indications for URS with retrograde effective use of flexible scopes/ suction facilities and for PCNL with miniaturization of the equipments used [2, 3]. Although both modalities are being performed in all parts of the world with successful outcomes in experienced hands, they are not completely safe where some possible well-known complications could occur at every step but particularly during post-operative period after these procedures [4–10].

Infection related complications are the most critical issue to be kept in mind prior to these minimal invasive procedures particularly before/during retrograde intrarenal manipulations. Although flexible ureteroscopic renal stone removal has become a valuable alternative on this aspect, application of the procedure for larger stones with acceptable stone free rates may increase the risk of such complications [3, 4].Accumulated experience and published data indicate that such complications (which sometimes could be lethal) need to be kept in mind with a very careful/rational treatment plan [8, 9, 11]. Infective problems after fURS procedures include post-operative fever occuring in 7.1%, UTI in 8.1% and sepsis in 5.4% of the cases managed [7–10]. In addition such infective complication could be encountered in 0.9–16.7% of the cases after percutaneous stone removal procedures [7–15].

In the light of these facts, to prevent such problems which could even be lethal in some cases, a rational and careful pre-operative diagnostic approach seems to be highly critical [24].The presence of any UTI should be well evaluated and treated prior to such interventions [16, 17, 20]. A systemic review showed that a urine culture test is superior to simple urine analysis test in ruling out bacteriuria and should therefore be performed in every candidate with symptoms of infection prior to endoscopic stone removal procedures [20].However, a negative urine dipstick test has also been found to be effective and predictive as a screening test with this aim [21].

In other words, it seems to be sufficient obtaining a pre-operative urine culture only in case of a positive urine analysis test regarding the measures to be taken for post-operative infective complications. In accordance to the recommendations of the EAU and the AUA, all patients need to receive an intraoperative single-shot antibiotic prophylaxis [5, 6, 10];except patients, who already received a sensitivity test-guided pre-operative antibiotic treatment based on a significant bacterial growth (≥ 10E5 CFU/ml) in urine culture. The systemic review and metanalysis of Lo et al. showed that a prophylactic antibiotic treatment can reduce the incidence of pyuria and bacteriuria following ureterorenoscopic laser lithotripsy, but not the incidence of clinically relevant UTI [16]. On the contrary, Gravas et al. showed, that patients undergoing PCNL without perioperative antibiotic prophylaxis had a significant higher rate of fever (*p* = 0.04) and complications within the first 30 days (< 0.0001) of post-operative period compared to patients undergoing antibiotic prophylaxis [18].

Thus, a well planned, rational decision making (evaluating the stone and patient related factors) along with a thorough urine examination seem to be the most crucial factors for safe and successful outcomes. In an attempt to limit or prevent such infective complications which may sometimes cause death after such endoscopic stone removal procedures, every candidate patient should be well evaluated for the presence and severity of UTI. Following the performance of a mid-stream urine culture and sensitivity test in case of a documented infection, UTI should inevitably be treated to render the patient infection free prior to such procedures. It is appropriate to obtain a urine culture for every patient where the presence of leucocytes and/or nitrite may be regarded as reliable indicators for a UTI. Related with this issue, the EAU recommends to perform a urinary microscopy and/or obtaining a urine culture before stone removal interventions for an effective antibiotic management [6]. However, both EAU and AUA guidelines acknowledge that their recommendations for antibiotic prophylaxis are based on limited evidence regarding the choice of antimicrobial agents, dose, timing, and duration of procedure [5, 6, 10].

On the other hand, no distinct data has been reported to date to emphasize the necessity of a perioperative antibioticprophylaxis for URS and this approach is being recommended by EAU guidelines to every patient undergoing endourological treatmentexcept simple diagnostic URS and distal ureteral stone treatment [16, 17]. Although the necessity and the effectivity of perioperative antibiotic prophylaxis were well evaluated in a limited number of studies so far with varying rate of success and degree of recommendations [18, 19], EAU guidelines state that, few studies could be derived from published literature defining the risk of infection following URS and percutaneous stone removal with no clear-cut evidence.

These findings emphasize that a careful evaluation regarding the presence of UTI and an appropriate management based on the culture sensitivity test outcomes seems to be highly critical before such interventions. With this rational approach, the likelihood of post-operative infection could be limited to a certain extent in such cases. A systematic review demonstrated that performance of a urine culture/sensitivity test is superior to a simple urinalysis in ruling out any infection and therefore this approach should be the standard in all patients[20]. However, although EAU guidelines recommend this approach; the exact need for an additional urine culture test after completion of antibiotic regimen before planned stone removal procedure to confirm the presence of a negative urine culture is not fully outlined with clinical evidence.

Evaluation of our findings showed no statistically significant difference regarding the rate of infective complications (presence of fever, incidence of septic findings), duration of hospitalization and readmission rates between two group of cases. Although the presence of a negative urine culture has been confirmed by urine culture test in group 1 cases, no additional urine culture was performed in group 2 cases (sterile urine was confirmed with only urinalysis) and the outcomes regarding the infective problems were found to be similiar.

In the light of our results and ongoing debate regarding the necessity of performing a second urine culture test following a sensitivity based antibiotic treatment before planned procedures; this approach seems to obtain a challenging status. Based on present our findings, elimination of a second urine culture test could bring the diagnostic procedures in a simplified status by lowering the laboratory workload and limit the overall costs. To the best of our knowledge, this is the first study which will in turn give reliable insights into this ongoing, challenging issue. Morover, this will let the endourologist to perform the procedure without any delay waiting for the result of second culture test (earlier removal of the stone and related problems) and more importantly let the patient have diminished stress with a well preserved quality of life.

Our study is not free of limitations. First of all the retrospective nature of the design is an important one and the number of cases included could be accepted as relatively small. However taking the highly limited data reported so far on this very critical issue into account, we believe that as the first trial focusing on this issue our data will be contributive enough on this aspect.

## Conclusion

Our current findings indicating no increased risk of infective complications in cases undergoing endoscopic stone removal procedures following the confirmation of negative urine culture with urinalysis only, emphasize well that a second urine culture test may not be a “ must” in these cases if the patients receive an appropriate antibiotic regimen based on the sensitivity test outcomes for a reasonable time period. However, we believe that further prospective randomized studies with larger series of cases focusing on this issue are certianly needed to support our findings.
